# Patterns of emergency admission for IBD patients over the last 10 years in Lothian, Scotland: a retrospective prevalent cohort analysis

**DOI:** 10.1111/apt.16867

**Published:** 2022-03-17

**Authors:** Mathew Lyons, Lauranne A. A. P. Derikx, James Fulforth, Sophie McCall, Nikolas Plevris, Philip W. Jenkinson, Kathryn Kirkwood, Spyros Siakavellas, Laura Lucaciu, Nathan Constantine‐Cooke, Ian D. Arnott, Paul Henderson, Richard K. Russell, David C. Wilson, Charlie W. Lees, Gareth‐Rhys Jones

**Affiliations:** ^1^ Edinburgh IBD Unit Western General Hospital Edinburgh UK; ^2^ Inflammatory Bowel Disease Center, Department of Gastroenterology and Hepatology Radboud University Medical Center Nijmegen the Netherlands; ^3^ Department of Gastroenterology Waikato District Health Board Hamilton New Zealand; ^4^ MRC Human Genetics Unit University of Edinburgh Edinburgh UK; ^5^ Centre for Genomic and Experimental Medicine University of Edinburgh Edinburgh UK; ^6^ Child Life and Health University of Edinburgh Edinburgh UK; ^7^ Department of Paediatric Gastroenterology and Nutrition Royal Hospital for Children and Young People Edinburgh UK; ^8^ Centre for Inflammation Research The Queen’s Medical Research Institute, University of Edinburgh Edinburgh UK

**Keywords:** epidemiology, infection, inflammatory bowel disease

## Abstract

**Objective:**

It is unclear how the compounding prevalence of inflammatory bowel disease (IBD) has translated into the causes and rates of hospitalisation, particularly in an era of increased biologic prescribing. We aimed to analyse these trends in a population‐based IBD cohort over the last 10 years.

**Design:**

The Lothian IBD registry is a complete, validated, prevalent database of IBD patients in NHS Lothian, Scotland. ICD‐10 coding of hospital discharge letters from all IBD patient admissions to secondary care between 1 January 2010 and 31 December 2019 was interrogated for admission cause, with linkage to local/national data sets on death and prescribed drugs.

**Results:**

Fifty‐seven per cent (4673/8211) of all IBD patients were admitted to secondary care for >24 h between 1 January 2010 and 31 December 2019. In patients <40 years, IBD was the commonest reason for admission (38% of admissions), whereas infection was the most common cause in those >60 years (19% of admissions). Three per cent (243/8211) of IBD patients accounted for 50% of the total IBD bed‐days over the study period.

Age‐standardised IBD admission rates fell from 39.4 to 25.5 admissions per 100,000 population between 2010 and 2019, an average annual percentage reduction of 3% (95% CI −4.5% to −2.1%, *p* < 0.0001). Non‐IBD admission rates were unchanged overall (145–137 per 100,000 population) and specifically for serious (hospitalisation) and severe (ITU admission or death) infection over the same period.

**Conclusion:**

Despite compounding prevalence and increased biologic use, IBD admission rates are falling. The cause of admission varies with age, with infection the predominant cause in older patients.

## Graphical abstract

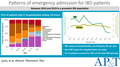

## 1 INTRODUCTION

Inflammatory bowel diseases (IBDs), comprising Crohn’s disease (CD), ulcerative colitis (UC) and IBD‐unclassified (IBD‐U), are chronic relapsing–remitting inflammatory conditions of the gastrointestinal tract. IBD is mostly diagnosed in younger people, is a lifelong and incurable disease, but without excess mortality.^1^ Due to incidence exceeding mortality by three to fourfold each year in the UK,^2^ the prevalence has increased from 0·3% in 2003 to 0·7% in 2018 and is projected to be >1% by 2028.^2^, ^3^ As a result of this compounding prevalence, we predict there will be more IBD patients in our cohort >50 years of age in 2028 than were in our entire prevalent cohort in 2018.^2^

Hospital admission is a common complication of IBD, estimated at 147/100,000 in 2003, rising to 353/100,000 in 2013,^4^ with hospitalisation the single biggest cost of IBD care in the prebiologic era.^5^ However, this does not account for non‐IBD causes for admission. Indeed, the relative contribution of IBD and non‐IBD reasons for hospitalisation are unknown.

Furthermore, there has been a paradigm shift in the therapy of IBD over the last 10 years, with biologic therapies increasingly given earlier in the disease course.^6^, ^7^ We have shown the impact of this in a prevalent IBD population with increased biologic prescribing and reduced surgery rates over time.^2^, ^6^, ^7^, ^8^ However, IBD patients are getting older with increased multimorbidity. It is unknown how these changes in treatment practice impact IBD hospital admission in number, rate and cause.

In contrast to ustekinumab and vedolizumab, anti‐TNF therapy is known to be associated with an increased risk of serious infection, defined as infection requiring hospitalisation.^9^, ^10^, ^11^ However, neither multivariable analysis of anti‐TNF use in 3000 CD patients nor meta‐analysis of over 5000 patients from controlled trials has revealed any increased risk of mortality from infection versus other IBD treatments or placebo, respectively.^10^, ^12^

The first aim of this study was to describe temporal changes in hospital admission rates, causes and outcomes in a prevalent IBD population between 2010 and 2019. Second, we aimed to describe both serious (hospitalisation) and severe (intensive therapy unit [ITU] admission or death) infection in hospitalised IBD patients.

## What is already known about this subject?

Hospitalisation for IBD is common but it is unclear how this is changing over time, particularly in the context of an ageing population and the more common use of immunomodulatory therapy. Similarly, it is not known what effects these shifts in population demographics and prescribing practices have had on the causes of hospitalisation (IBD and non‐IBD) among IBD patients.

## What are the new findings?

We show, in a prevalent population over 10 years, that over half of all IBD patients were hospitalised and that age‐standardised IBD hospitalisation has fallen across all adult age groups, but non‐IBD hospitalisation rates are static. The principal non‐IBD cause for admission was an infection. The risk of hospitalisation for infection increased with age such that it was the most common reason for admission overall in patients >60 years.

## How might it impact clinical practice?

The median age of people living with IBD in the Western world is increasing due to compounding prevalence. We will increasingly see older IBD patients hospitalised for non‐IBD causes, particularly infection, with significant implications for service provision, continuity of care and treatment decision‐making.

## 2 METHODS

### 2.1 Study population

NHS Lothian provides universal, free‐at‐point of care, healthcare for 907,580 people (mid‐2019 population estimate) covering a geographically defined area of 1724 km^2^ (Edinburgh city, Mid Lothian, West Lothian and East Lothian) of which 77.8% identify as White Scottish ethnicity.^13^, ^14^, ^15^ Secondary care is delivered by four hospitals (The Western General Hospital, The Royal Infirmary of Edinburgh, St John’s Hospital and The Royal Hospital for Sick Children of Edinburgh). Every person in Scotland has a unique Community Health Index (CHI) number to identify residents for healthcare purposes. This permits robust linkage of patient records to a range of detailed national registries. The Lothian IBD registry (LIBDR) was compiled through a capture–recapture methodology, describes the observed prevalence of IBD in Lothian between 2008 and 2018, and has been maintained as a prospective registry since 1 August 2018.^2^

In this initial work, IBD cases were identified using a variety of hospital and primary care sources including hospital admission coding, pathology coding, prescribing databases and existing registries. The electronic patient record for all 'possible' cases was reviewed and the diagnosis was confirmed using standard criteria. Prevalent IBD cases between 1 January 2010 and 31 December 2019 were therefore identified using a combination of LIBDR‐derived date of diagnosis and linkage to mortality registries and residency (postcode).

### 2.2 Data collection

The electronic patient records for all IBD patients in the Lothian IBD Registry were interrogated for IBD subtype, clinic attendance, hospital admission(s) and demographic data. Admission body mass index data for adults were defined as underweight (<18), normal (18–24.9), overweight (25–29.9) or obese (≥30) from the closest available height and weight records to the date of admission. Data were analysed on a year‐by‐year basis with the patient age calculated as (year of analysis) − (year of birth) and point prevalence for each calendar year calculated on 31 December.

### 2.3 Hospital admissions

All in‐patient hospital discharge letters in NHS Lothian have been coded using the international classification of diseases codes (ICD‐10) since 1 August 2006. Each discharge letter contains a primary diagnosis code identifying the main diagnosis pertaining to the admission as well as any number of secondary diagnosis codes. Diagnoses are manually entered by the treating clinician, thereafter the most appropriate ICD‐10 code is ascribed by administrative staff. The ICD‐10 'major' code accuracy for primary diagnosis codes for NHS Lothian hospitals is between 93% and 99%.^16^, ^17^

All primary diagnosis codes for admissions (both elective and unscheduled) from 1 January 2010 to 31 December 2019 for all patients with a confirmed diagnosis of IBD were obtained. Two schemas were used to group admission reasons. For overall groupings (termed 'super‐groups'), ICD‐10 chapters were applied with the following modifications:

Chapter I (Certain infectious and parasitic diseases) was integrated with infections arising from other bodily systems (e.g. I40.0 Infective Myocarditis) (Table S1).

To identify broad system‐based admission causes, ICD‐10 codes from Chapter XVIII (Symptoms, signs, and abnormal clinical and laboratory findings, not elsewhere classified) to Chapter XXI (Factors influencing health status and contact with health services) were, where possible, grouped under chapters of the relevant system (e.g. Chapter XVIII code R10 Abdominal and pelvic pain was grouped with Chapter XI—Diseases of the digestive system, see Table S2).

Diagnosis codes for Crohn’s disease (K50) and ulcerative colitis (K51) were extracted from 'Chapter XI—Diseases of the digestive system' and presented separately to allow direct comparison and were termed 'IBD‐related admissions'. Other admissions were classified as non‐IBD.

For the second schema, ICD‐10 major codes were used to identify diagnosis causes within super‐groups (e.g. Malignant Neoplasm of the Breast [C50] within the Neoplasms [Chapter II] super‐group).

In infection severity analyses, secondary admission diagnosis coding for infection was also included. Hospital admissions <24 h duration were excluded from the admission cause analysis to remove elective day‐case attendance (e.g. biologic infusion, endoscopy). Admissions that occurred within 60 days of the IBD diagnosis date were also excluded.

### 2.4 Primary care prescribing

Primary care prescribing in Scotland has been universally electronic since 2009. It has been audited by the NHS Scotland Information Services Division and coded using the British National Formulary (BNF) hierarchy.^18^ The database contains all items dispensed in Scotland. It does not include items prescribed that are not dispensed and does not include any medicines prescribed and dispensed within hospitals. Data for all BNF codes pertaining to thiopurines, opioids, steroids and antibiotics were obtained (Table S3). A primary care prescription was termed relevant to admission if it was dispensed within 90 days before the date of hospital admission.

### 2.5 Secondary care prescribing

All biologic therapy is prescribed in secondary care in NHS Lothian and details of all patients prescribed biologic therapy are prospectively recorded in the Lothian IBD Biologics Database, which is linked to the Lothian IBD Registry. Data on the drug, start date and stop date were linked to admissions and primary care prescribing data. Biologic therapies were termed relevant to admission if the last infusion was within 60 days before the date of hospital admission.

### 2.6 Cause of death

The cause of death for all deaths in Scotland is recorded on the medical certificate of the cause of death, a legal document completed by a senior clinician with knowledge of the patient’s condition prior to death. The medical certificate of the cause of death records the primary cause(s) of death along with any contributing or secondary causes and these data are held electronically by NHS Scotland Information Services Division, coded by ICD‐10 code. Cause(s) and date of death for all patients in the Lothian IBD Registry were sourced and linked by CHI number.

### 2.7 Predictors of admission with severe infection

A subset of admissions data where the infection was the primary or secondary reason for admission was created to establish predictors of intensive therapy unit admission or death due to infection (collectively termed 'severe infection') following hospital admission. All deaths, where an ICD‐10 code for infection (Table S1), appeared on the medical certificate of the cause of death and the death occurred either during admission for an infection or within 30 days of discharge (to account for discharges for end‐of‐life care) from admission for infection were included.

### 2.8 Statistical analysis

Data are reported according to the Strengthening the Reporting of Observational Studies in Epidemiology (STROBE) statements. Descriptive statistics are described as median with an interquartile range (non‐parametric). National Records Scotland publish yearly, mid‐year, single year of age population estimates for each Scottish health board area from 0 to 90 years of age.^13^ Annual population estimates for each age grouping were enumerated from this data and used as the denominator to calculate age‐standardised statistics per 100,000 population. Similarly, a denominator of the prevalent IBD population was used to calculate statistics per 1000 IBD patients. Temporal trends in hospitalisation rates were analysed using Poisson regression to calculate the annual average percentage change. Summary statistics (e.g. age) for the whole cohort were calculated at the end of the study period or on the date of death unless otherwise specified. Proportions were compared with *z* tests of proportions. Rates were compared using a negative binomial distribution to give a standard error of the difference. All tests were two‐tailed with a *p* value of <0.05 considered significant.

A logistic regression model was used to explore risk factors for severe infection. Categorical and binary variables for age, sex, IBD subtype, primary care prescribing, biologic prescribing, active disease (faecal calprotectin >250 μg/g) and BMI were investigated using increasing model complexity. Variables, their definitions and assumptions are shown in Table S7.

All analysis was conducted in R (version 4·0·3, R Foundation for Statistical Computing) using the tidyverse and finalfit packages.^19^, ^20^

### 2.9 Ethical approvals and patient involvement

The project was performed under the auspices of service assessment and was approved by the local Caldicott Guardian (Project ID: CRD18002, registered NHS Lothian information asset #IAR‐954). Patients or the public were not involved in the design, conduct, reporting or dissemination plans of our research.

## 3 RESULTS

### 3.1 Study population

A total of 8211 patients with IBD (3000 patients with CD, 4446 with UC and 765 with IBD‐U) were included in the analysis, with a median age of 55 years (IQR 40–71) and a median disease duration of 12 years (IQR 6–19) on 31 December 2019.

### 3.2 Hospital admissions

#### 3.2.1 All Admissions

Fifty‐seven per cent (4673/8211) of IBD patients were admitted to secondary care for >24 h between 1 January 2010 and 31 December 2019 totaling 16,195 admissions. Overall, these admissions accounted for 160,501 bed‐days, with a median admission duration of 4 days (IQR 2–9). There were 158/16,195 admissions (1%) where no diagnoses were assigned, due to missing coding, recent admission not yet coded, or a patient still in hospital at the end of the study period.

The commonest individual super‐group reason for admission overall was 'Diseases of the Digestive System (Chapter XI)' accounting for 41% (6606/16,195) of admissions. The top four major codes within this super‐group were 'Crohn’s disease (K50)' (11%, 1843/16,195), 'ulcerative colitis (K51)' (10%, 1589/16,195), 'Abdominal and pelvic pain (R10)' (4%, 627/16,195) and 'Paralytic ileus and intestinal obstruction without hernia (K56)' (3%, 417/16,195).

**FIGURE 1:**
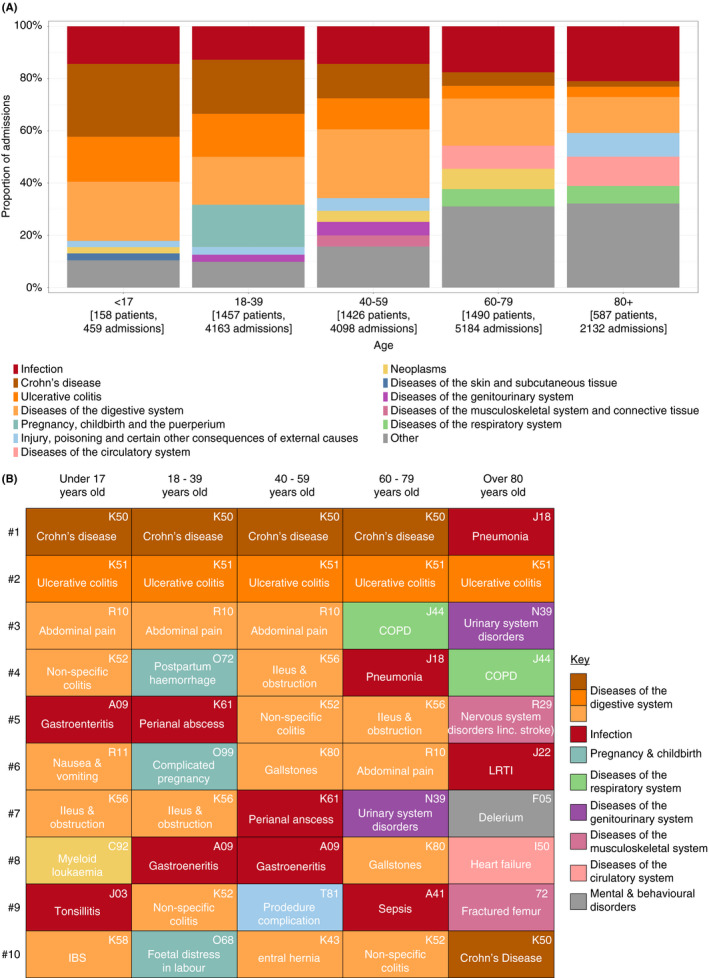
Primary cause for hospital admission in IBD patients between 1 January 2010 and 31 December 2019. ICD‐10 codes from hospital discharge letters were assessed for primary cause for admission, grouped by both ICD‐10 'super‐group' (Table S2) and ICD 'major' code, and stratified by age. (A) A minimum of seven top super‐group causes per age group are shown, plus CD and UC if not in the top seven, with 'other' admission super‐groups amalgamated and (B) the top 10 ICD‐10 major code admission reasons for each age group over the 10‐year period. Each cell is colour‐coded to correspond to the super‐group colour in (A). COPD, chronic obstructive pulmonary disease; IBS, irritable bowel syndrome; LRTI, lower respiratory tract infection

The next most common super‐group was infection (16%, 2549/16,195), which is considered in detail below (Figure 1, Table S4). The third and fourth most common super‐groups were 'Diseases of the circulatory system (Chapter IX)' (5%, 857/16,195) and 'Injury, poisoning and certain other consequences of external causes (Chapter XIX)' (5%, 809/16,195). Within these, the top major codes were 'Acute myocardial infarction (I21)' (1%, 133/16,195), 'Heart failure (I50)' (0·5%, 86/16,195) and Atrial fibrillation and flutter (I48) (0.5%, 84/16,195) for Chapter IX and Complications of other internal prosthetic devices, implants, and grafts (T85) (0·5%, 94/16,195), 'Fracture of femur (S72)' (0·5%, 91/16,195) and 'Complications of procedures, not elsewhere classified (T81)' (0·5%, 81/16,195) for Chapter XIX.

The cause for admission varied by age, with IBD the predominant cause in patients <40 years (Figure 1A). The proportion of all admissions due to CD fell with advancing age from 28% (541/4143) in those <17 years to 0.2% in >80 years (45/2152) (Figure 1B). With advancing age, not only did the causes for admission become more diverse (e.g. 5% of admissions >80 years were due to delirium) but also admission with infection became more common (20% of admissions >60 years) (Figure 1A).

#### 3.2.2 IBD admissions

**FIGURE 2:**
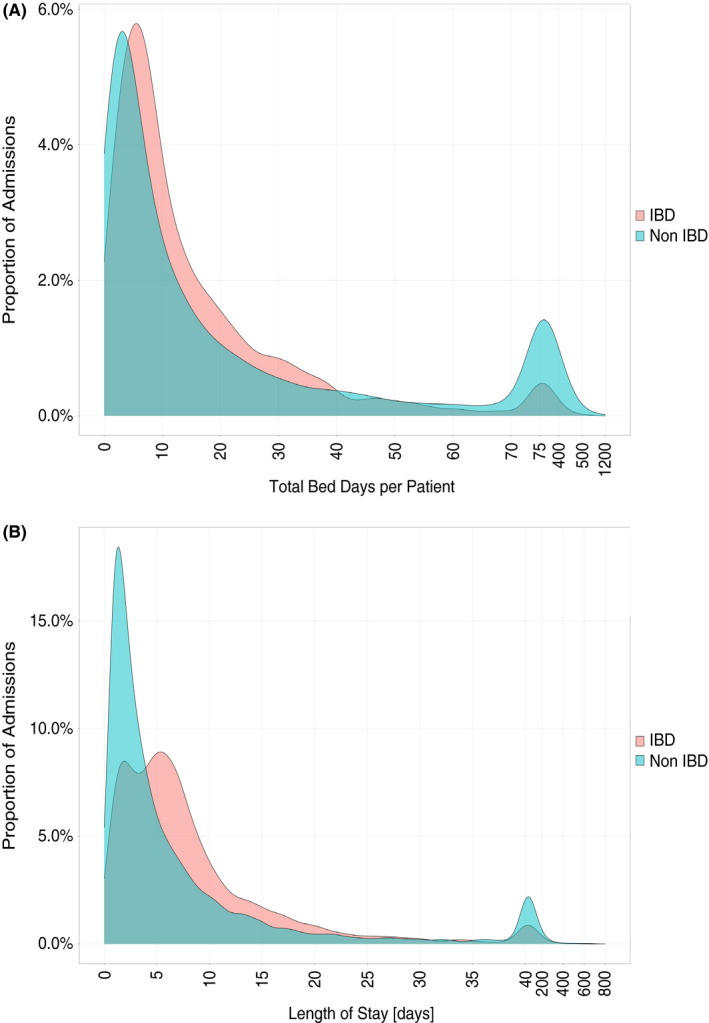
Distribution of length of stay for all hospital admissions stratified by IBD (K50/K51) or non‐IBD cause for admission. (A) Total aggregate days in the hospital and (B) length of individual hospital stays for each patient admitted over the 10‐year period

Overall, 21% (3432/16,195) of all admissions (1810 patients) were primarily due to IBD. These admissions accounted for 31,729 bed‐days, with a median individual length of stay of 6 days (IQR 3–10) (Figure 2A), and a median number of IBD‐related admissions per patient of 1 (IQR 1–2, maximum 31).

A subset of the cohort demonstrated 'high‐healthcare usage', disproportionately contributing to hospital admissions, whereby 3% (243/8211) of patients accounted for 50% of total IBD bed‐days (Figure 2A). The median age at first admission within the study period for this group was 40 years (26–56), 44% were male (108/243), 47% CD, 48% UC and 4% IBD‐U, with a median duration of disease of 9 years at the end of the study (IQR 5–15). Regarding treatment, 26% (62/243) of the group had failed two or more biologic drugs with a 72% and 81% chance of being treated with an opiate or steroid over the 10‐year period, respectively, compared with 34% and 44% in non‐high healthcare users.

**FIGURE 3:**
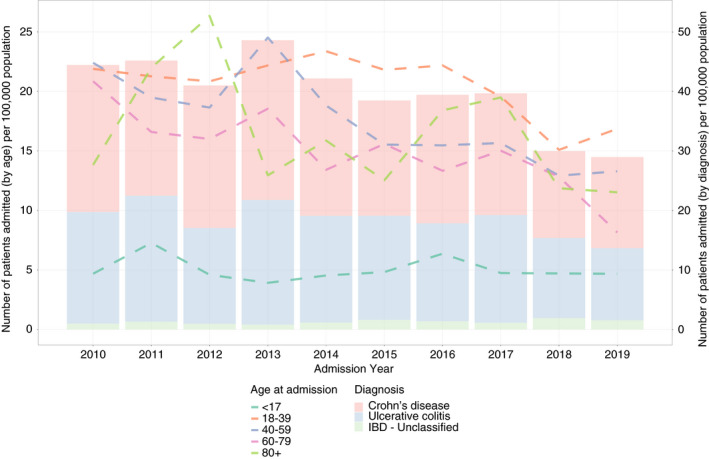
Age‐standardised IBD‐related hospital admission rates stratified by age and IBD subtype between 1 January 2010 and 31 December 2019

Age‐standardised IBD admission rates overall have fallen from 39.4/100,000 in 2010 to 25.5/100,000 in 2019, a decrease of 36% overall (Table 1, Figure 3) and an average annual percentage change of −3% (95% CI −4.5% to −2.1%, *p* < 0.0001). All adult age groups showed declining age‐standardised IBD admission rates over this period (Figure 3). In contrast, admission rates for non‐IBD‐related causes were static in all age groups (145–137 admission/100,000 population from 2010 to 2019, average annual percentage change of −0.4% [95% CI −2% to 1%, *p* = 0.6]) (Figure S1).

**TABLE 1:** Annual IBD‐related hospital admissions are shown as the number of patients admitted, number admitted per 100,000 population in NHS Lothian and number admitted per 1000 IBD patients in NHS Lothian. Mid‐year population estimates for the NHS Lothian Healthboard area are produced on an annual basis^13^

Year	Patients admitted annually	Population estimate of NHS Lothian	IBD population	Admissions per 100,000 population	Admissions per 1000 IBD patients
2010	325	825,520	5222	39.4	62.2
2011	322	836,610	5495	38.5	58.6
2012	298	843,740	5769	35.3	51.7
2013	337	849,720	6000	39.7	56.2
2014	302	858,120	6282	35.2	48.1
2015	289	867,800	6531	33.3	44.3
2016	302	880,000	6728	34.3	44.9
2017	288	889,450	6960	32.4	41.4
2018	231	897,770	7006	25.7	33.0
2019	231	907,580	7091	25.5	32.6

Following discharge from the hospital after an admission for IBD (3432 admissions), there were 157 (5%) readmissions within 7 days, 276 (8%) within 14 days and 461 (13%) within 30 days. In 49% of cases, the reason for readmission was also listed as IBD. In the remaining cases, readmission was for 'Infection (Table S1)' (13%), 'Abdominal and pelvic pain (R10)' (5%) or 'Paralytic ileus and intestinal obstruction without hernia (K56)' (4%).

#### 3.2.3 Infection admissions

Sixteen per cent of all admissions (2539/16,195) in 1550 patients had a primary diagnosis of infection (Table S4), with an additional 1708 admissions in 531 patients where the admission was complicated by infection (secondary diagnosis). Therefore, 26% of all admissions (4247/16,195) in 2081 patients were primarily for, or complicated by, infection.

Respiratory (33%, 1398/4247), urinary tract (21%, 901/4247) and gastrointestinal infections (29%, 1248/4247) accounted for 84% (3547/4247) of infection or infection‐complicated admissions. Three per cent of infections (135/4247) were complicated by bacteriemia. The commonest pathogens were coagulase‐negative *Staphylococcus* (24%), *Escherichia coli* (15%) and *Staphylococcus epidermidis* (9%).

The type of infection changed with age. Gastrointestinal sources were the most common in patients <40 years, accounting for approximately 40% of infection admissions each year. In patients over >60 years, however, respiratory infections were more common, accounting for 40–50% of infection admissions (Figure S2).

#### 3.2.4 Admissions with severe infection

Severe infection (ITU admission or death) was observed in 5% (193/4247) of IBD patients admitted with infection over the 10 years of study, with approximately 2 severe infections per 100,000 persons each year (Table 2). There was no significant change in severe infection rates over the duration of the study (average annual percentage change of 1.6% [95% CI −3.2% to 6.6%, *p* = 0.54]). There were 100 admissions to ITU for infection for 85 patients (Table S5) and 107 deaths within 30 days of admission where the infection was listed as a primary or secondary cause on the death certificate (Table S6). Multivariable logistic regression analysis revealed, aside from age, that there were no significant predictors of severe infection.

**TABLE 2:** Annual severe infection rates shown as the number of patients admitted to ITU or dying due to infection per 100,000 population and 1000 IBD patients in NHS Lothian

Year	Patients with severe infection	Population estimate of NHS Lothian	IBD population	Severe infection per 100,000 population	Severe infection per 1000 IBD patients
2010	21	825,520	5195	2.5	4.0
2011	20	836,610	5467	2.4	3.7
2012	15	843,740	5739	1.8	2.6
2013	12	849,720	5967	1.4	2.0
2014	20	858,120	6249	2.3	3.2
2015	17	867,800	6495	2.0	2.6
2016	28	880,000	6690	3.2	4.2
2017	18	889,450	6922	2.0	2.6
2018	24	897,770	6968	2.7	3.4
2019	18	907,580	7047	2.0	2.6

## 4 DISCUSSION

In this work, we describe the causes of hospital admission in a prevalent IBD population over 10 years, incorporating >30,000 admissions and >175,000 days in the hospital.

Age‐standardised admission rates for IBD have fallen significantly over a period during which there have been substantial increases in biologic use.^6^, ^7^ Reassuringly, we have not observed an increase in the risk of severe infection (ITU admission or death) in IBD patients between 2010 and 2020.

The 10‐year hospital admission risk for IBD patients in the entire prevalent cohort was 57%. The median length of stay of 4 days (median 6 days for IBD admissions, 3 days for non‐IBD admissions). We found that IBD patients >80 years had similar age‐standardised IBD admission rates to younger adults but higher age‐standardised non‐IBD admission rates. Interestingly, the total number of IBD bed‐days were disproportionately attributed to a small fraction of the cohort, with 3% of prevalent patients accounting for 50% of the total, the majority of whom had significant opiate, steroid and biologic exposure. This disproportionate use of healthcare resources in a small fraction of the IBD population demands further in‐depth study, to enable preventative strategies to be developed and introduced.

We have shown that age‐standardised IBD admission rates overall have fallen irrespective of IBD subtype or age. The exception was in those <17 years, where we observed constant admission rates, in keeping with previous data.^21^ In contrast, non‐IBD admission rates in all age groups were static. When combined with an ageing population, this trend suggests non‐IBD causes will become the dominant reason for IBD patient admission in the years to come.

To our knowledge, there are no previous temporal analyses of all‐cause IBD patient hospitalisation. The most recent comparative UK IBD admission data from Ahmad et al. using Hospital Episode Statistics (HES) analyses showed striking increases in IBD admissions between 2003 and 2013.^4^ However, the driver for this increase was predominately changes in elective admissions, with emergency IBD admissions only rising slightly from 26–37 to 49–65/100,000 for UC and CD, respectively.

Infection admission rates in our data were static over time and strongly correlated to age. In addition to serious infection, defined as an infection that requires hospital admission, we have reported further temporal data regarding infection severity stratified by ITU admission or death (severe infection). Multivariable logistic regression analysis revealed no significant predictors of severe infection (aside from age). However, due to the low number of events, particularly in biologic‐ and steroid‐treated groups, we do not feel that our data set is powered to answer this question robustly.

Indeed, biologic use in older patients remains an important issue. A recent meta‐analysis of 14 studies of biologic initiation for IBD, rheumatoid arthritis or psoriasis in older patients (>60 years) showed that there was a threefold increased risk of any reported infection (i.e. including non‐hospitalised infections) versus older patients not exposed to biologics, but no effect on all‐cause mortality.^22^ However, undertreating older patients is also a concern, with older incident CD patients more likely to be treated with steroids and less likely to be prescribed biologics than younger patients, despite a higher risk of surgery.^23^

The strength of our study lies in the completeness of the underlying cohort. The LIBDR is derived by capture–recapture methodologies and is 94.3% complete.^2^ With linkage by CHI number across other data sets, we have presented a complete description of the burden of IBD on secondary care in a large, urban area, where the ICD‐10 major category coding accuracy is >94%.^16^ Historically, these data have been limited by difficulties identifying the true prevalence of disease, especially those with limited involvement with secondary care. As a result, we report for the first time population‐level changes in all‐cause admission rates for IBD patients over time.

Our study is limited by the lack of detailed data about disease phenotype and disease activity. In addition, it was not possible from these data to ascertain whether an admission was planned, (i.e. elective) or unscheduled, thus we used duration of admission as a proxy to remove short elective admissions. Although it is attractive to speculate falling admission rates might be due to improvements in our use of IBD therapy, we cannot ascribe causality to this retrospective data set. Furthermore, we cannot exclude that these observations are due to a change in our diagnosis of IBD, for example, in milder cases due to increased public/physician awareness of the condition over the study period. We were also not able to assess levels of community infection in IBD patients and thus were unable to use this as a control group to assess risk factors for serious (i.e. hospitalised) infection. Last, a complete list of patient comorbidities is not routinely captured in hospital discharge coding. Thus, the effect of comorbidity in an ageing IBD population and its effect on hospital admission is an important area for future work.

## 5 CONCLUSION

We report the first temporal analysis of all‐cause IBD patient admission in a prevalent population. Fifty‐seven per cent of all IBD patients in NHS Lothian were admitted at least once, for at least 24 h, within a 10‐year period. IBD admission rates, but not non‐IBD admission rates, have fallen significantly over the last 10 years. Diseases of the digestive system, and in particular IBD, were the commonest all‐age cause for admission. This was followed by infection, which increased progressively with age to become the commonest cause for admission in those >60 years.

## AUTHOR CONTRIBUTIONS

**Mathew Lyons:** Conceptualisation (equal); data curation (lead); formal analysis (lead); methodology (lead); project administration (lead); writing – original draft (lead); writing – review and editing (lead). **Lauranne A. A. P. Derikx:** Data curation (supporting); methodology (supporting); writing – original draft (supporting); writing – review and editing (supporting). **James Fulforth:** Data curation (supporting); writing – original draft (supporting); writing – review and editing (supporting). **Sophie McCall:** Data curation (equal); writing – original draft (supporting); writing – review and editing (supporting). **Nikolas Plevris:** Conceptualisation (supporting); data curation (equal); formal analysis (supporting); methodology (supporting); writing – original draft (supporting); writing – review and editing (supporting). **Philip W Jenkinson:** Data curation (supporting); methodology (supporting); writing – original draft (supporting); writing – review and editing (supporting). **Kate Kirkwood:** Data curation (supporting); writing – original draft (supporting); writing – review and editing (supporting). **Spyros I Siakavellas:** Conceptualisation (supporting); data curation (supporting); methodology (supporting); writing – original draft (supporting); writing – review and editing (supporting). **Laura Lucaciu:** Writing – original draft (supporting); writing – review and editing (supporting). **Nathan Constantine‐Cooke:** Data curation (supporting); formal analysis (supporting); methodology (supporting); writing – original draft (supporting); writing – review and editing (supporting). **Ian Arnott:** Supervision (supporting); writing – original draft (supporting); writing – review and editing (supporting). **Paul Henderson:** Conceptualisation (supporting); data curation (supporting); methodology (supporting); project administration (supporting); writing – original draft (supporting); writing – review and editing (supporting). **Richard Russell:** Conceptualisation (supporting); data curation (supporting); methodology (supporting); writing – original draft (supporting); writing – review and editing (supporting). **David C Wilson:** Conceptualisation (supporting); data curation (supporting); methodology (supporting); supervision (supporting); writing – original draft (supporting); writing – review and editing (supporting). **Charles W Lees:** Conceptualisation (equal); investigation (equal); methodology (equal); project administration (equal); supervision (equal); writing – original draft (equal); writing – review and editing (equal). **Gareth‐Rhys Jones:** Conceptualisation (lead); data curation (equal); formal analysis (equal); investigation (equal); methodology (equal); project administration (equal); supervision (lead); writing – original draft (equal); writing – review and editing (equal).

## Supporting information


Figure S1
Figure S2
Table S1
Table S2
Table S3
Table S4
Table S5
Table S6
Table S7

## Data Availability

Data is not available to be shared due to the granular nature making even an anonymised data set identifiable by other means (e.g. admission reason).
