# Supplementary material for: Patterns of emergency admission for IBD patients over the last 10 years in Lothian, Scotland: a retrospective prevalent cohort analysis
Source: Aliment Pharmacol Ther. 2022 Mar 17;56(1):67–76. doi: 10.1111/apt.16867 (PMC9314623; doi:10.1111/apt.16867)
Supplement: Supplementary file 1 — Figure S1 Figure S2 Table S1 Table S2 Table S3 Table S4 Table S5 Table S6 Table S7 [file APT-56-67-s001.docx]

**Supplementary Appendix**

**Contents**

Supplementary Figure 1. 2

Supplementary Figure 2 3

Supplementary Table 1 4

Supplementary Table 2 16

Supplementary Table 3 18

Supplementary Table 4 19

Supplementary Table 5. 20

Supplementary Table 6 21

Supplementary Table 7 22

# Supplementary Figure 1. Age standardised non-IBD-cause hospital admission rates for IBD patients by IBD subtype between 01/01/2010 and 31/12/2019.


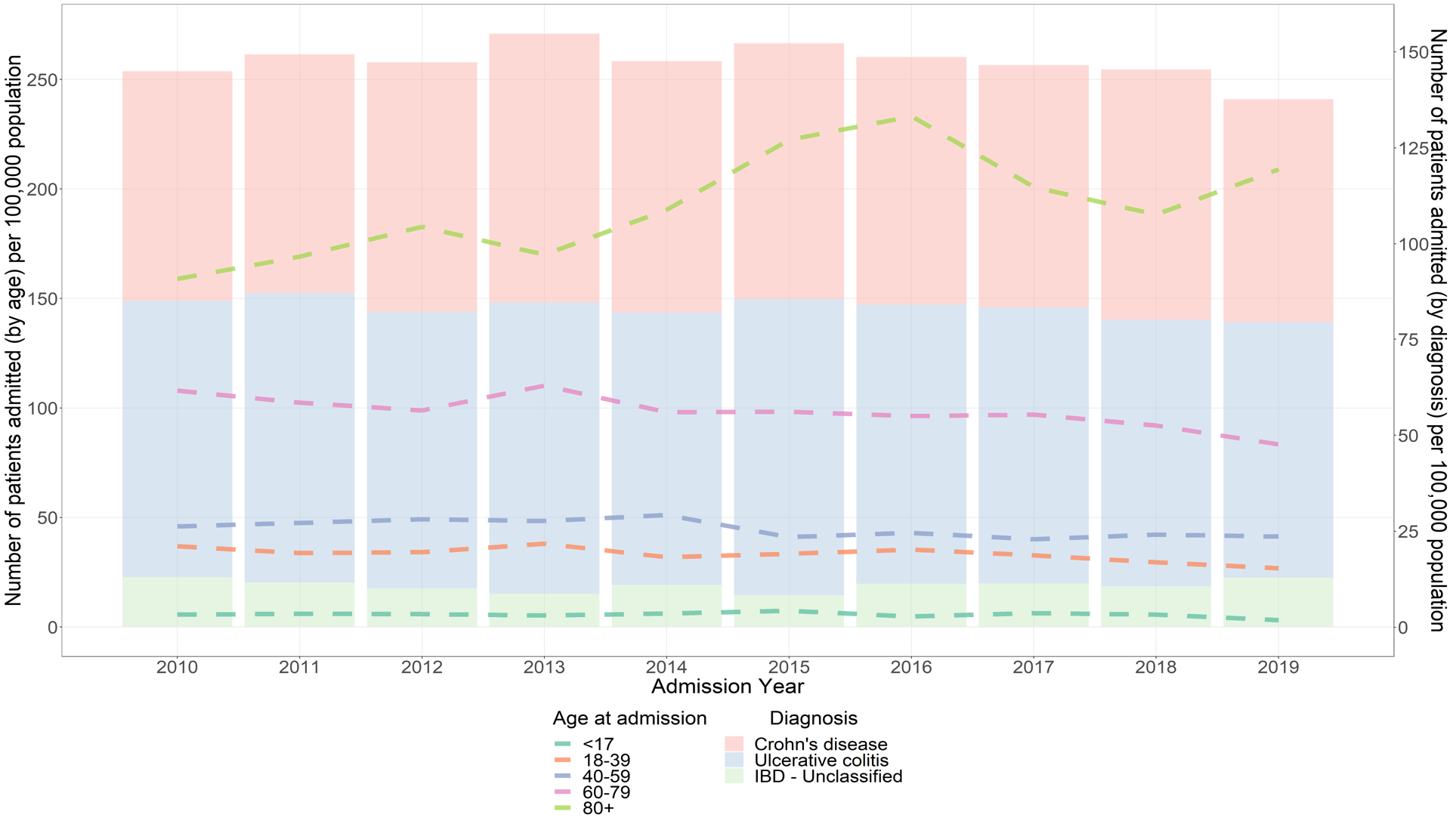


# Supplementary Figure 2. Cause of Infection admission stratified by age and time (01/01/2010 and 31/12/2019).


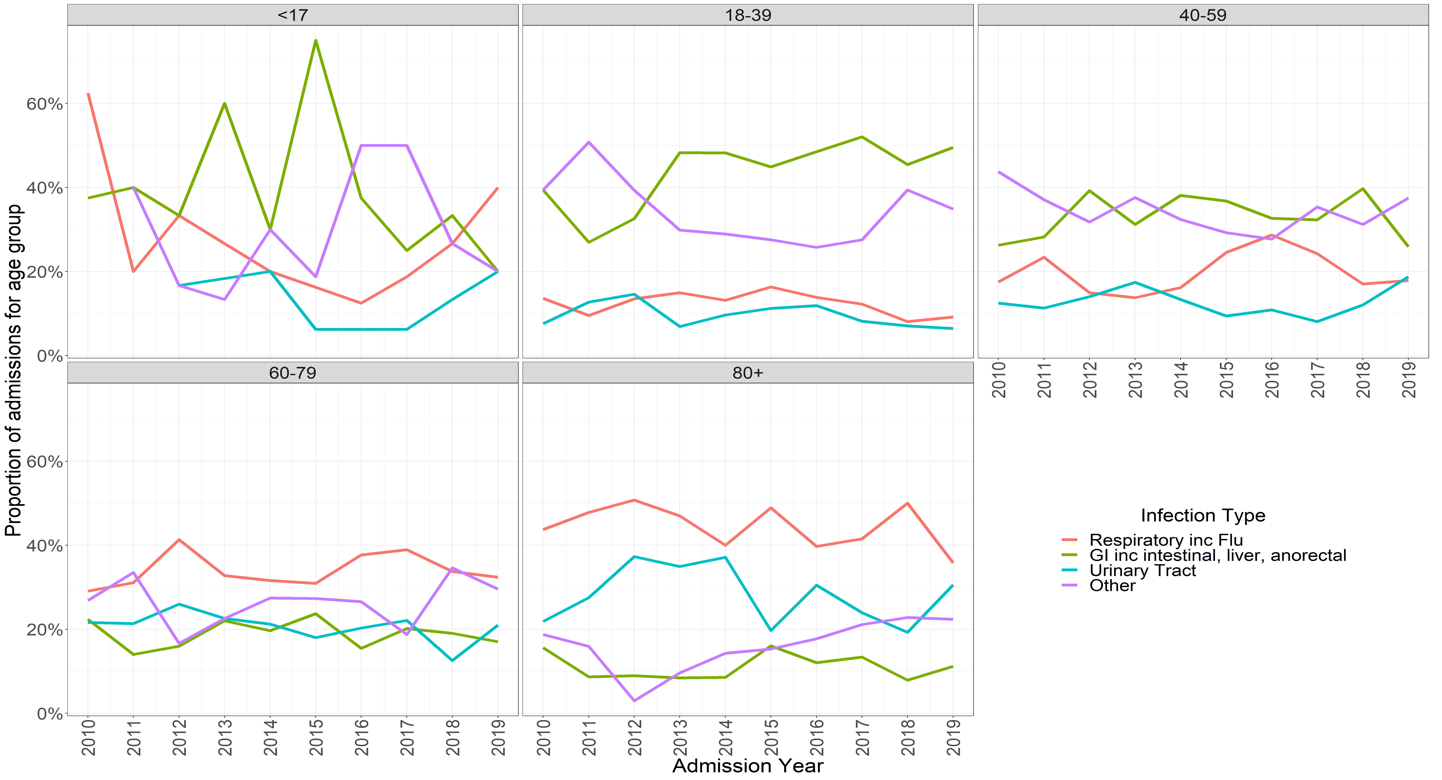


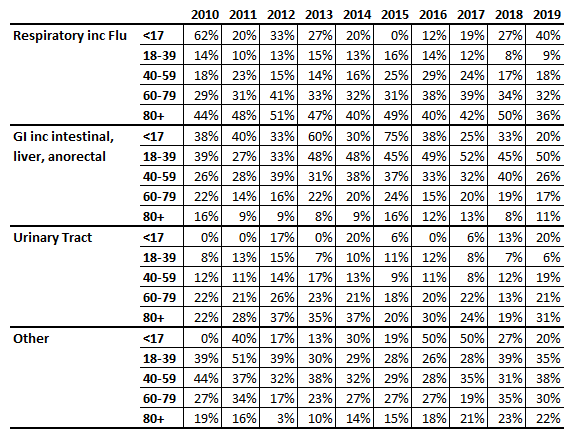


# Supplementary Table 1. ICD-10 codes categorised as “Infection”

| **ICD Code** | **Description** |
| --- | --- |
| A00 | Cholera |
| A01 | Typhoid and paratyphoid fevers |
| A01.1 | Paratyphoid fever A |
| A02 | Other salmonella infections |
| A02.0 | Salmonella enteritis |
| A02.9 | Salmonella infection, unspecified |
| A03 | Shigellosis |
| A03.3 | Shigellosis due to Shigella sonnei |
| A04 | Other bacterial intestinal infections |
| A04.1 | Enterotoxigenic Escherichia coli infection |
| A04.4 | Other intestinal Escherichia coli infections |
| A04.5 | Campylobacter enteritis |
| A04.7 | Enterocolitis due to Clostridium difficile |
| A04.8 | Other specified bacterial intestinal infections |
| A04.9 | Bacterial intestinal infection, unspecified |
| A05 | Other bacterial foodborne intoxications, not elsewhere classified |
| A05.9 | Bacterial foodborne intoxication, unspecified |
| A06 | Amoebiasis |
| A06.0 | Acute amoebic dysentery |
| A07 | Other protozoal intestinal diseases |
| A07.1 | Giardiasis [lambliasis] |
| A07.2 | Cryptosporidiosis |
| A08 | Viral and other specified intestinal infections |
| A08.0 | Rotaviral enteritis |
| A08.1 | Acute gastroenteropathy due to Norwalk agent |
| A08.3 | Other viral enteritis |
| A08.4 | Viral intestinal infection, unspecified |
| A09 | Other gastroenteritis and colitis of infectious and unspecified origin |
| A09.0 | Other and unspecified gastroenteritis and colitis of infectious origin |
| A09.9 | Gastroenteritis and colitis of unspecified origin |
| A15 | Respiratory tuberculosis, bacteriologically and histologically confirmed |
| A16 | Respiratory tuberculosis, not confirmed bacteriologically or histologically |
| A17 | Tuberculosis of nervous system |
| A18 | Tuberculosis of other organs |
| A19 | Miliary tuberculosis |
| A20 | Plague |
| A21 | Tularaemia |
| A22 | Anthrax |
| A23 | Brucellosis |
| A24 | Glanders and melioidosis |
| A25 | Rat-bite fevers |
| A26 | Erysipeloid |
| A27 | Leptospirosis |
| A28 | Other zoonotic bacterial diseases, not elsewhere classified |
| A30 | Leprosy [Hansen disease] |
| A31 | Infection due to other mycobacteria |
| A32 | Listeriosis |
| A36 | Diphtheria |
| A37 | Whooping cough |
| A39 | Meningococcal infection |
| A40 | Streptococcal sepsis |
| A40.8 | Other streptococcal sepsis |
| A40.9 | Streptococcal sepsis, unspecified |
| A41 | Other sepsis |
| A41.0 | Sepsis due to Staphylococcus aureus |
| A41.1 | Sepsis due to other specified staphylococcus |
| A41.5 | Sepsis due to other Gram-negative organisms |
| A41.8 | Other specified sepsis |
| A41.9 | Sepsis, unspecified |
| A42 | Actinomycosis |
| A43 | Nocardiosis |
| A44 | Bartonellosis |
| A46 | Erysipelas |
| A48 | Other bacterial diseases, not elsewhere classified |
| A48.1 | Legionnaires disease |
| A49 | Bacterial infection of unspecified site |
| A49.0 | Staphylococcal infection, unspecified site |
| A49.1 | Streptococcal and enterococcal infection, unspecified site |
| A49.8 | Other bacterial infections of unspecified site |
| A50 | Congenital syphilis |
| A51 | Early syphilis |
| A52 | Late syphilis |
| A53 | Other and unspecified syphilis |
| A54 | Gonococcal infection |
| A55 | Chlamydial lymphogranuloma (venereum) |
| A56 | Other sexually transmitted chlamydial diseases |
| A57 | Chancroid |
| A58 | Granuloma inguinale |
| A59 | Trichomoniasis |
| A60 | Anogenital herpesviral [herpes simplex] infection |
| A60.0 | Herpesviral infection of genitalia and urogenital tract |
| A63 | Other predominantly sexually transmitted diseases, not elsewhere classified |
| A75 | Typhus fever |
| A77 | Spotted fever [tick-borne rickettsioses] |
| A79 | Other rickettsioses |
| A81 | Atypical virus infections of central nervous system |
| A87.2 | Lymphocytic choriomeningitis |
| A87.9 | Viral meningitis, unspecified |
| A89 | Unspecified viral infection of central nervous system |
| A99 | Unspecified viral haemorrhagic fever |
| B00.0 | Eczema herpeticum |
| B00.3 | Herpesviral meningitis |
| B01.9 | Varicella without complication |
| B02.2 | Zoster with other nervous system involvement |
| B02.3 | Zoster ocular disease |
| B02.8 | Zoster with other complications |
| B02.9 | Zoster without complication |
| B17.2 | Acute hepatitis E |
| B17.9 | Acute viral hepatitis, unspecified |
| B20.3 | HIV disease resulting in other viral infections |
| B20.6 | HIV disease resulting in Pneumocystis jirovecii pneumonia |
| B20.7 | HIV disease resulting in multiple infections |
| B20.8 | HIV disease resulting in other infectious and parasitic diseases |
| B22.7 | HIV disease resulting in multiple diseases classified elsewhere |
| B23.8 | HIV disease resulting in other specified conditions |
| B25.8 | Other cytomegaloviral diseases |
| B25.9 | Cytomegaloviral disease, unspecified |
| B27 | Infectious mononucleosis |
| B27.0 | Gammaherpesviral mononucleosis |
| B27.9 | Infectious mononucleosis, unspecified |
| B34.0 | Adenovirus infection, unspecified site |
| B34.8 | Other viral infections of unspecified site |
| B34.9 | Viral infection, unspecified |
| B37.7 | Candidal sepsis |
| B37.9 | Candidiasis, unspecified |
| B44.1 | Other pulmonary aspergillosis |
| B44.9 | Aspergillosis, unspecified |
| B55.0 | Visceral leishmaniasis |
| B59 | Pneumocystosis |
| G00 | Bacterial meningitis, not elsewhere classified |
| G01 | Meningitis in bacterial diseases classified elsewhere |
| G02 | Meningitis in other infectious and parasitic diseases classified elsewhere |
| G04.2 | Bacterial meningoencephalitis and meningomyelitis, not elsewhere classified |
| G04.9 | Encephalitis, myelitis and encephalomyelitis, unspecified |
| G05 | Encephalitis, myelitis and encephalomyelitis in diseases classified elsewhere |
| G06 | Intracranial and intraspinal abscess and granuloma |
| G07 | Intracranial and intraspinal abscess and granuloma in diseases classified elsewhere |
| H00 | Hordeolum and chalazion |
| H05.0 | Acute inflammation of orbit |
| H10.0 | Mucopurulent conjunctivitis |
| H10.8 | Other conjunctivitis |
| H44.0 | Purulent endophthalmitis |
| H60.0 | Abscess of external ear |
| H60.1 | Cellulitis of external ear |
| H60.2 | Malignant otitis externa |
| H60.3 | Other infective otitis externa |
| H60.9 | Otitis externa, unspecified |
| H62.0 | Otitis externa in bacterial diseases classified elsewhere |
| H62.1 | Otitis externa in viral diseases classified elsewhere |
| H62.2 | Otitis externa in mycoses |
| H65 | Nonsuppurative otitis media |
| H65.2 | Chronic serous otitis media |
| H65.3 | Chronic mucoid otitis media |
| H66 | Suppurative and unspecified otitis media |
| H66.9 | Otitis media, unspecified |
| H67 | Otitis media in diseases classified elsewhere |
| H70 | Mastoiditis and related conditions |
| I30.1 | Infective pericarditis |
| I32.0 | Pericarditis in bacterial diseases classified elsewhere |
| I32.1 | Pericarditis in other infectious and parasitic diseases classified elsewhere |
| I33 | Acute and subacute endocarditis |
| I38 | Endocarditis, valve unspecified |
| I39 | Endocarditis and heart valve disorders in diseases classified elsewhere |
| I40.0 | Infective myocarditis |
| I41.0 | Myocarditis in bacterial diseases classified elsewhere |
| I41.1 | Myocarditis in viral diseases classified elsewhere |
| I41.2 | Myocarditis in other infectious and parasitic diseases classified elsewhere |
| J00 | Acute nasopharyngitis [common cold] |
| J01 | Acute sinusitis |
| J02 | Acute pharyngitis |
| J02.0 | Streptococcal pharyngitis |
| J02.8 | Acute pharyngitis due to other specified organisms |
| J03 | Acute tonsillitis |
| J03.9 | Acute tonsillitis, unspecified |
| J04 | Acute laryngitis and tracheitis |
| J04.0 | Acute laryngitis |
| J04.1 | Acute tracheitis |
| J05 | Acute obstructive laryngitis [croup] and epiglottitis |
| J05.1 | Acute epiglottitis |
| J06 | Acute upper respiratory infections of multiple and unspecified sites |
| J06.9 | Acute upper respiratory infection, unspecified |
| J09 | Influenza due to identified zoonotic or pandemic influenza virus |
| J10 | Influenza due to identified seasonal influenza virus |
| J10.0 | Influenza with pneumonia, seasonal influenza virus identified |
| J10.1 | Influenza with other respiratory manifestations, seasonal influenza virus identified |
| J11 | Influenza, virus not identified |
| J11.0 | Influenza with pneumonia, virus not identified |
| J11.1 | Influenza with other respiratory manifestations, virus not identified |
| J12 | Viral pneumonia, not elsewhere classified |
| J12.0 | Adenoviral pneumonia |
| J12.1 | Respiratory syncytial virus pneumonia |
| J12.3 | Human metapneumovirus pneumonia |
| J12.8 | Other viral pneumonia |
| J13 | Pneumonia due to Streptococcus pneumoniae |
| J14 | Pneumonia due to Haemophilus influenzae |
| J15 | Bacterial pneumonia, not elsewhere classified |
| J15.0 | Pneumonia due to Klebsiella pneumoniae |
| J15.1 | Pneumonia due to Pseudomonas |
| J15.2 | Pneumonia due to staphylococcus |
| J15.3 | Pneumonia due to streptococcus, group B |
| J15.4 | Pneumonia due to other streptococci |
| J15.5 | Pneumonia due to Escherichia coli |
| J15.6 | Pneumonia due to other Gram-negative bacteria |
| J15.7 | Pneumonia due to Mycoplasma pneumoniae |
| J16 | Pneumonia due to other infectious organisms, not elsewhere classified |
| J17 | Pneumonia in diseases classified elsewhere |
| J18 | Pneumonia, organism unspecified |
| J18.0 | Bronchopneumonia, unspecified |
| J18.1 | Lobar pneumonia, unspecified |
| J18.9 | Pneumonia, unspecified |
| J20 | Acute bronchitis |
| J21 | Acute bronchiolitis |
| J21.8 | Acute bronchiolitis due to other specified organisms |
| J21.9 | Acute bronchiolitis, unspecified |
| J22 | Unspecified acute lower respiratory infection |
| J32 | Chronic sinusitis |
| J34.0 | Abscess, furuncle and carbuncle of nose |
| J36 | Peritonsillar abscess |
| J39.0 | Retropharyngeal and parapharyngeal abscess |
| J39.1 | Other abscess of pharynx |
| J69 | Pneumonitis due to solids and liquids |
| J69.0 | Pneumonitis due to food and vomit |
| J85 | Abscess of lung and mediastinum |
| J86 | Pyothorax |
| K04.6 | Periapical abscess with sinus |
| K04.7 | Periapical abscess without sinus |
| K11.3 | Abscess of salivary gland |
| K12.2 | Cellulitis and abscess of mouth |
| K35 | Acute appendicitis |
| K35.2 | Acute appendicitis with generalized peritonitis |
| K35.3 | Acute appendicitis with localized peritonitis |
| K35.8 | Acute appendicitis, other and unspecified |
| K37 | Unspecified appendicitis |
| K61 | Abscess of anal and rectal regions |
| K61.0 | Anal abscess |
| K61.1 | Rectal abscess |
| K61.2 | Anorectal abscess |
| K61.3 | Ischiorectal abscess |
| K61.4 | Intrasphincteric abscess |
| K63.0 | Abscess of intestine |
| K65 | Peritonitis |
| K65.8 | Other peritonitis |
| K65.9 | Peritonitis, unspecified |
| K67.0 | Chlamydial peritonitis |
| K67.1 | Gonococcal peritonitis |
| K67.2 | Syphilitic peritonitis |
| K67.3 | Tuberculous peritonitis |
| K75.0 | Abscess of liver |
| K80.0 | Calculus of gallbladder with acute cholecystitis |
| K80.1 | Calculus of gallbladder with other cholecystitis |
| K80.3 | Calculus of bile duct with cholangitis |
| K80.4 | Calculus of bile duct with cholecystitis |
| K81 | Cholecystitis |
| K81.0 | Acute cholecystitis |
| K81.1 | Chronic cholecystitis |
| K81.9 | Cholecystitis, unspecified |
| K83.0 | Cholangitis |
| L01 | Impetigo |
| L02 | Cutaneous abscess, furuncle and carbuncle |
| L02.0 | Cutaneous abscess, furuncle and carbuncle of face |
| L02.1 | Cutaneous abscess, furuncle and carbuncle of neck |
| L02.2 | Cutaneous abscess, furuncle and carbuncle of trunk |
| L02.3 | Cutaneous abscess, furuncle and carbuncle of buttock |
| L02.4 | Cutaneous abscess, furuncle and carbuncle of limb |
| L03 | Cellulitis |
| L03.0 | Cellulitis of finger and toe |
| L03.1 | Cellulitis of other parts of limb |
| L03.2 | Cellulitis of face |
| L03.3 | Cellulitis of trunk |
| L03.8 | Cellulitis of other sites |
| L03.9 | Cellulitis, unspecified |
| L04 | Acute lymphadenitis |
| L05 | Pilonidal cyst |
| L08 | Other local infections of skin and subcutaneous tissue |
| L08.9 | Local infection of skin and subcutaneous tissue, unspecified |
| M00 | Pyogenic arthritis |
| M01 | Direct infections of joint in infectious and parasitic diseases classified elsewhere |
| M46.2 | Osteomyelitis of vertebra |
| M60.0 | Infective myositis |
| M65.0 | Abscess of tendon sheath |
| M65.00 | Abscess of tendon sheath |
| M65.01 | Abscess of tendon sheath |
| M65.02 | Abscess of tendon sheath |
| M65.03 | Abscess of tendon sheath |
| M65.04 | Abscess of tendon sheath |
| M65.05 | Abscess of tendon sheath |
| M65.06 | Abscess of tendon sheath |
| M65.07 | Abscess of tendon sheath |
| M65.08 | Abscess of tendon sheath |
| M65.09 | Abscess of tendon sheath |
| M65.1 | Other infective (teno)synovitis |
| M65.10 | Other infective (teno)synovitis |
| M65.11 | Other infective (teno)synovitis |
| M65.12 | Other infective (teno)synovitis |
| M65.13 | Other infective (teno)synovitis |
| M65.14 | Other infective (teno)synovitis |
| M65.15 | Other infective (teno)synovitis |
| M65.16 | Other infective (teno)synovitis |
| M65.17 | Other infective (teno)synovitis |
| M65.18 | Other infective (teno)synovitis |
| M65.19 | Other infective (teno)synovitis |
| M71.0 | Abscess of bursa |
| M71.00 | Abscess of bursa |
| M71.01 | Abscess of bursa |
| M71.02 | Abscess of bursa |
| M71.03 | Abscess of bursa |
| M71.04 | Abscess of bursa |
| M71.05 | Abscess of bursa |
| M71.06 | Abscess of bursa |
| M71.07 | Abscess of bursa |
| M71.08 | Abscess of bursa |
| M71.09 | Abscess of bursa |
| M71.1 | Other infective bursitis |
| M71.10 | Other infective bursitis |
| M71.11 | Other infective bursitis |
| M71.12 | Other infective bursitis |
| M71.13 | Other infective bursitis |
| M71.14 | Other infective bursitis |
| M71.15 | Other infective bursitis |
| M71.16 | Other infective bursitis |
| M71.17 | Other infective bursitis |
| M71.18 | Other infective bursitis |
| M71.19 | Other infective bursitis |
| M72.6 | Necrotizing fasciitis |
| M72.60 | Necrotizing fasciitis |
| M72.61 | Necrotizing fasciitis |
| M72.62 | Necrotizing fasciitis |
| M72.63 | Necrotizing fasciitis |
| M72.64 | Necrotizing fasciitis |
| M72.65 | Necrotizing fasciitis |
| M72.66 | Necrotizing fasciitis |
| M72.67 | Necrotizing fasciitis |
| M72.68 | Necrotizing fasciitis |
| M72.69 | Necrotizing fasciitis |
| M73.0 | Gonococcal bursitis |
| M73.00 | Gonococcal bursitis |
| M73.01 | Gonococcal bursitis |
| M73.02 | Gonococcal bursitis |
| M73.03 | Gonococcal bursitis |
| M73.04 | Gonococcal bursitis |
| M73.05 | Gonococcal bursitis |
| M73.06 | Gonococcal bursitis |
| M73.07 | Gonococcal bursitis |
| M73.08 | Gonococcal bursitis |
| M73.09 | Gonococcal bursitis |
| M73.1 | Syphilitic bursitis |
| M73.10 | Syphilitic bursitis |
| M73.11 | Syphilitic bursitis |
| M73.12 | Syphilitic bursitis |
| M73.13 | Syphilitic bursitis |
| M73.14 | Syphilitic bursitis |
| M73.15 | Syphilitic bursitis |
| M73.16 | Syphilitic bursitis |
| M73.17 | Syphilitic bursitis |
| M73.18 | Syphilitic bursitis |
| M73.19 | Syphilitic bursitis |
| M86 | Osteomyelitis |
| M86.67 | Other chronic osteomyelitis |
| M86.85 | Other osteomyelitis |
| M86.9 | Osteomyelitis, unspecified |
| M86.95 | Osteomyelitis, unspecified |
| M86.97 | Osteomyelitis, unspecified |
| M90.0 | Tuberculosis of bone |
| M90.00 | Tuberculosis of bone |
| M90.01 | Tuberculosis of bone |
| M90.02 | Tuberculosis of bone |
| M90.03 | Tuberculosis of bone |
| M90.04 | Tuberculosis of bone |
| M90.05 | Tuberculosis of bone |
| M90.06 | Tuberculosis of bone |
| M90.07 | Tuberculosis of bone |
| M90.08 | Tuberculosis of bone |
| M90.09 | Tuberculosis of bone |
| N13.6 | Pyonephrosis |
| N15.1 | Renal and perinephric abscess |
| N16.0 | Renal tubulo-interstitial disorders in infectious and parasitic diseases classified elsewhere |
| N30.0 | Acute cystitis |
| N30.2 | Other chronic cystitis |
| N30.8 | Other cystitis |
| N30.9 | Cystitis, unspecified |
| N33.0 | Tuberculous cystitis |
| N34 | Urethritis and urethral syndrome |
| N39.0 | Urinary tract infection, site not specified |
| N41 | Inflammatory diseases of prostate |
| N41.2 | Abscess of prostate |
| N43.1 | Infected hydrocele |
| N45 | Orchitis and epididymitis |
| N45.0 | Orchitis, epididymitis and epididymo-orchitis with abscess |
| N45.9 | Orchitis, epididymitis and epididymo-orchitis without abscess |
| N70 | Salpingitis and oophoritis |
| N70.9 | Salpingitis and oophoritis, unspecified |
| N71 | Inflammatory disease of uterus, except cervix |
| N73 | Other female pelvic inflammatory diseases |
| N73.9 | Female pelvic inflammatory disease, unspecified |
| N74.1 | Female tuberculous pelvic inflammatory disease |
| N74.2 | Female syphilitic pelvic inflammatory disease |
| N74.3 | Female gonococcal pelvic inflammatory disease |
| N74.4 | Female chlamydial pelvic inflammatory disease |
| N75.1 | Abscess of Bartholin gland |
| N76.0 | Acute vaginitis |
| N76.1 | Subacute and chronic vaginitis |
| N76.2 | Acute vulvitis |
| N76.3 | Subacute and chronic vulvitis |
| N76.4 | Abscess of vulva |
| O07.0 | Failed medical abortion, complicated by genital tract and pelvic infection |
| O07.5 | Other and unspecified failed attempted abortion, complicated by genital tract and pelvic infection |
| O08.0 | Genital tract and pelvic infection following abortion and ectopic and molar pregnancy |
| O23 | Infections of genitourinary tract in pregnancy |
| O23.3 | Infections of other parts of urinary tract in pregnancy |
| O23.4 | Unspecified infection of urinary tract in pregnancy |
| O23.9 | Other and unspecified genitourinary tract infection in pregnancy |
| O75.3 | Other infection during labour |
| O85 | Puerperal sepsis |
| O86 | Other puerperal infections |
| O86.4 | Pyrexia of unknown origin following delivery |
| O86.8 | Other specified puerperal infections |
| O91 | Infections of breast associated with childbirth |
| O91.2 | Nonpurulent mastitis associated with childbirth |
| O98 | Maternal infectious and parasitic diseases classifiable elsewhere but complicating pregnancy, childbirth and the puerperium |
| T81.4 | Infection following a procedure, not elsewhere classified |
| T82.6 | Infection and inflammatory reaction due to cardiac valve prosthesis |
| T82.7 | Infection and inflammatory reaction due to other cardiac and vascular devices, implants and grafts |
| T83.5 | Infection and inflammatory reaction due to prosthetic device, implant and graft in urinary system |
| T84.5 | Infection and inflammatory reaction due to internal joint prosthesis |
| T85.7 | Infection and inflammatory reaction due to other internal prosthetic devices, implants and grafts |

# Supplementary Table 2. List of ICD-10 “major” codes removed from their original chapters and joined with chapters as listed in order to create “super-groups” for grouping admissions. ICD-10 codes not listed were included in the “super-group” pertaining to their ICD-10 defined chapter, except for codes related to infectious pathology as in Supplementary Table 1.

| **ICD-10 “Major” Code** | **New chapter for “super-group”** |
| --- | --- |
| Respiratory tuberculosis, not confirmed bacteriologically or histologically [A16] | Chapter X |
| Vitamin B12 deficiency anaemia [D51] | Chapter XVIII |
| Other disorders of pancreatic internal secretion [E16] | Chapter XI |
| Diseases of thymus [E32] | Chapter III |
| Unspecified severe protein-energy malnutrition [E43X] | Chapter XVIII |
| Unspecified protein-energy malnutrition [E46X] | Chapter XVIII |
| Vitamin D deficiency [E55] | Chapter XVIII |
| Deficiency of other nutrient elements [E61] | Chapter XVIII |
| Obesity [E66] | Chapter XI |
| Disorders of mineral metabolism [E83] | Chapter XVIII |
| Amyloidosis [E85] | Chapter XIII |
| Volume depletion [E86X] | Chapter XVIII |
| Other disorders of fluid, electrolyte and acid-base balance [E87] | Chapter XVIII |
| Other metabolic disorders [E88] | Chapter XI |
| Haemorrhoids [I84] | Chapter XI |
| Dentofacial anomalies [including malocclusion] [K07] | Chapter XIII |
| Other diseases of jaws [K10] | Chapter XIII |
| Other perinatal digestive system disorders [P78] | Chapter XVI |
| Abnormalities of heart beat [R00] | Chapter IX |
| Haemorrhage from respiratory passages [R04] | Chapter X |
| Cough [R05X] | Chapter X |
| Abnormalities of breathing [R06] | Chapter X |
| Abdominal and pelvic pain [R10] | Chapter XI |
| Nausea and vomiting [R11X] | Chapter XI |
| Dysphagia [R13X] | Chapter XI |
| Flatulence and related conditions [R14X] | Chapter XI |
| Faecal incontinence [R15X] | Chapter XI |
| Unspecified jaundice [R17X] | Chapter XI |
| Ascites [R18X] | Chapter XI |
| Other symptoms and signs involving the digestive system and abdomen [R19] | Chapter XI |
| Disturbances of skin sensation [R20] | Chapter XII |
| Rash and other nonspecific skin eruption [R21X] | Chapter XII |
| Other skin changes [R23] | Chapter XII |
| Abnormalities of gait and mobility [R26] | Chapter XIII |
| Other lack of coordination [R27] | Chapter VI |
| Pain associated with micturition [R30] | Chapter XIV |
| Unspecified haematuria [R31X] | Chapter XIV |
| Unspecified urinary incontinence [R32X] | Chapter XIV |
| Retention of urine [R33X] | Chapter XIV |
| Polyuria [R35X] | Chapter XIV |
| Other symptoms and signs involving the urinary system [R39] | Chapter XIV |
| Somnolence, stupor and coma [R40] | Chapter VI |
| Other symptoms and signs involving cognitive functions and awareness [R41] | Chapter V |
| Dizziness and giddiness [R42X] | Chapter VI |
| Symptoms and signs involving emotional state [R45] | Chapter V |
| Headache [R51X] | Chapter VI |
| Malaise and fatigue [R53X] | Chapter V |
| Senility [R54X] | Chapter V |
| Syncope and collapse [R55X] | Chapter VI |
| Convulsions, not elsewhere classified [R56] | Chapter VI |
| Haemorrhage, not elsewhere classified [R58X] | Chapter IX |
| Lack of expected normal physiological development [R62] | Chapter VI |
| Symptoms and signs concerning food and fluid intake [R63] | Chapter XI |
| Other abnormal findings of blood chemistry [R79] | Chapter III |
| Abnormal findings in specimens from digestive organs and abdominal cavity [R85] | Chapter XI |
| Abnormal findings on diagnostic imaging of central nervous system [R90] | Chapter VI |
| Abnormal findings on diagnostic imaging of lung [R91X] | Chapter X |
| Follow-up examination after treatment for malignant neoplasms [Z08] | Chapter II |
| Follow-up examination after treatment for conditions other than malignant neoplasms [Z09] | Chapter II |
| Supervision of normal pregnancy [Z34] | Chapter XV |
| Antenatal screening [Z36] | Chapter XV |
| Postpartum care and examination [Z39] | Chapter XV |
| Other orthopaedic follow-up care [Z47] | Chapter XIII |

# Supplementary Table 3. BNF Prescription Codes

| **BNF Chapter** | **BNF Section** | **BNF Paragraph** |
| --- | --- | --- |
| 4 Central Nervous System | 407 Analgesics | 40702 Opioid Analgesics |
| 5 Infections | 501 Antibacterial Drugs | 50101 Penicillins |
| 5 Infections | 501 Antibacterial Drugs | 50105 Macrolides |
| 5 Infections | 501 Antibacterial Drugs | 50108 Sulfonamides And Trimethoprim |
| 5 Infections | 501 Antibacterial Drugs | 50102 Cephalosporins and other Beta-Lactams |
| 5 Infections | 501 Antibacterial Drugs | 50103 Tetracyclines |
| 5 Infections | 501 Antibacterial Drugs | 50112 Quinolones |
| 5 Infections | 501 Antibacterial Drugs | 50111 Metronidazole, Tinidazole & Ornidazole |
| 5 Infections | 501 Antibacterial Drugs | 50113 Urinary-Tract Infections |
| 5 Infections | 501 Antibacterial Drugs | 50109 Antituberculosis Drugs |
| 5 Infections | 501 Antibacterial Drugs | 50107 Some Other Antibacterials |
| 5 Infections | 501 Antibacterial Drugs | 50106 Clindamycin and Lincomycin |
| 5 Infections | 501 Antibacterial Drugs | 50110 Antileprotic Drugs |
| 5 Infections | 501 Antibacterial Drugs | 50104 Aminoglycosides |
| 6 Endocrine System | 603 Corticosteroids (Endocrine) | 60302 Glucocorticoid Therapy |
| 8 Malignant Disease & Immunosuppression | 802 Drugs Affecting The Immune Response | 80201 Antiproliferative Immunosuppressants |
| 8 Malignant Disease & Immunosuppression | 801 Cytotoxic Drugs | 80103 Antimetabolites |

# Supplementary Table 4. Admission reasons by “super-group”

| **Admission Reason** | **Proportion of admissions** |
| --- | --- |
| Diseases of the digestive system [Chapter XI] | 20% |
| Infection [See supplementary table 1] | 16% |
| Crohn’s Disease [K50] | 11% |
| Ulcerative Colitis [K51] | 10% |
| Diseases of the circulatory system [Chapter IX] | 5% |
| Injury, poisoning and certain other consequences of external causes [Chapter XIX] | 5% |
| Neoplasms [Chapter II] | 5% |
| Diseases of the genitourinary system [Chapter XIV] | 5% |
| Pregnancy, childbirth and the puerperium [Chapter XV] | 4% |
| Diseases of the musculoskeletal system and connective tissue [Chapter XIII] | 4% |
| Diseases of the respiratory system [Chapter X] | 4% |
| Symptoms, signs and abnormal clinical and laboratory findings, not elsewhere classified [Chapter XVIII] | 3% |
| Mental, Behavioral and Neurodevelopmental disorders [Chapter V] | 2% |
| Diseases of the nervous system [Chapter VI] | 2% |
| Factors influencing health status and contact with health services [Chapter XXI] | 2% |
| Diseases of the blood and blood-forming organs and certain disorders involving the immune mechanism [Chapter III] | 1% |
| Diseases of the skin and subcutaneous tissue [Chapter XII] | 1% |
| Diseases of the eye and adnexa [Chapter VII] | 1% |
| Endocrine, nutritional and metabolic diseases [Chapter IV] | <1% |
| Diseases of the ear and mastoid process [Chapter VIII] | <1% |
| Congenital malformations, deformations and chromosomal abnormalities [Chapter XVII] | <1% |
| Certain conditions originating in the perinatal period [Chapter XVI] | <1% |

# Supplementary Table 5. Number of admissions to ITU for each infection type. Other includes urinary tract infection, dermatological infections (eg cellulitis), viral infections (eg CMV, EBV, HIV), musculoskeletal infections, infections of the reproductive system, unspecified bacterial infections and unspecified infections following a procedure.

| **Infection Type** | **Number of admissions** |
| --- | --- |
| Respiratory inc Flu | 37 |
| GI inc intestinal, liver, anorectal | 24 |
| Sepsis (aetiology not recorded) | 23 |
| Other | 16 |

# Supplementary Table 6. Number of deaths for each infection type. Total is greater than the total number of deaths as some deaths were attributed to more than one infection on the MCCD. Other includes dermatological infections (eg cellulitis), viral infections (eg CMV, EBV, HIV), musculoskeletal infections, peritonitis, infections involving the heart, unspecified bacterial infections, and unspecified infections following a procedure.

| **Infection Type** | **Number of deaths attributed** |
| --- | --- |
| Respiratory inc Flu | 77 |
| Sepsis (aetiology not recorded) | 16 |
| GI inc intestinal, liver, anorectal | 12 |
| Urinary Tract | 9 |
| Other | 17 |

# Supplementary Table 7. Variables investigated as potential risk factors for severe infection

| **Variable** | **Type** | **Definition** | **Rational/Notes** |
| --- | --- | --- | --- |
| Age | Categorical | Young (<39) Mid age (40-69) Elderly (>70) | Splitting to categorical is more interpretable than a continuous variable |
| Sex | Binary | M or F | - |
| Thiopurines | Binary | Drug of this class dispensed from a community prescription in the 90 days before admission | 90-day cut off as many patients get 3 months of medications dispensed at a time on repeat |
| Steroids | Binary | Drug of this class dispensed from a community prescription in the 90 days before admission | 90-day cut off as many patients get 3 months of medications dispensed at a time on repeat |
| Opioids | Binary | Drug of this class dispensed from a community prescription in the 90 days before admission | 90-day cut off as many patients get 3 months of medications dispensed at a time on repeat |
| Biologics | Binary | Biologic administered in the 60 days before admission | 60 days will cover the maximum interval between doses of biologics. If last biologic was >60 days ago it may have been stopped but not yet recorded in the database. |
